# Two distinct modes of nucleosome modulation associated with different degrees of dependence of nucleosome positioning on the underlying DNA sequence

**DOI:** 10.1186/1471-2164-10-15

**Published:** 2009-01-10

**Authors:** Zhiming Dai, Xianhua Dai, Qian Xiang, Jihua Feng, Jiang Wang, Yangyang Deng, Caisheng He

**Affiliations:** 1 Electronic Department, Sun Yat-Sen University, Guangzhou, PR China

## Abstract

**Background:**

The nucleosome is the fundamental unit of eukaryotic genomes. Its positioning plays a central role in diverse cellular processes that rely on access to genomic DNA. Experimental evidence suggests that the genomic DNA sequence is one important determinant of nucleosome positioning. Yet it is less clear whether the role of the underlying DNA sequence in nucleosome positioning varies across different promoters. Whether different determinants of nucleosome positioning have characteristic influences on nucleosome modulation also remains to be elucidated.

**Results:**

We identified two typical promoter classes in yeast associated with high or low dependence of nucleosome positioning on the underlying DNA sequence, respectively. Importantly, the two classes have low or high intrinsic sequence preferences for nucleosomes, respectively. The two classes are further distinguished by multiple promoter features, including nucleosome occupancy, nucleosome fuzziness, H2A.Z occupancy, changes in nucleosome positions before and after transcriptional perturbation, and gene activity. Both classes have significantly high turnover rates of histone H3, but employ distinct modes of nucleosome modulation: The first class is characterized by hyperacetylation, whereas the second class is highly regulated by ATP-dependent chromatin remodelling.

**Conclusion:**

Our results, coupled with the known features of nucleosome modulation, suggest that the two distinct modes of nucleosome modulation selectively employed by different genes are linked with the intrinsic sequence preferences for nucleosomes. The difference in modes of nucleosome modulation can account for the difference in the contribution of DNA sequence to nucleosome positioning between both promoter classes.

## Background

The majority of the DNA in eukaryotic cells is packaged into nucleosomes. The nucleosome is composed of a histone octamer around which 147 DNA base pairs are wrapped [1]. Nucleosome positioning plays an essential role in diverse cellular processes by controlling accessibility of genomic DNA to regulatory factors [2]. In general, there are three main ways in which cells regulate nucleosomal influences on these cellular processes: chromatin remodeling [3], histone modification [4], and incorporation of histone variants [5]. High-resolution nucleosome positions across genomes have been identified in yeast (Saccharomyces cerevisiae) [6-11]. These valuable data make it possible to understand how nucleosome positions are exactly determined in vivo.

The coordination of nucleosome positions is a complex process involving combined interactions among multiple factors. Experimental evidence indicates that certain DNA sequences have strong ability to bend and twist [12]. Consequently, DNA sequences differ greatly in their ability to wrap around histones. The binding affinities can vary by several orders of magnitude [13]. Recent studies have used DNA sequence features to predict genome-wide nucleosome positions with modest success [14-18], confirming that nucleosome positioning is partially encoded in the genomic DNA sequence. Yet it is less clear whether the underlying DNA sequence plays a uniform role in nucleosome positioning throughout the genome. Until recently, two studies have observed decay in contribution of the underlying DNA sequence to nucleosome positioning along the coding region [9,10]. On the other hand, while recent results collectively suggest that factors besides DNA sequence preferences also contribute to nucleosome positioning [8,19], they leave open the question of whether different determinants of nucleosome positioning have distinct influences on nucleosome regulation.

In this study, we investigated into the relationship between the degree of dependence of nucleosome positioning on the underlying DNA sequence and the mode of nucleosome modulation in yeast. We identified two typical promoter classes associated with high or low dependence of nucleosome positioning on the underlying DNA sequence, respectively. The two classes are distinguished by multiple promoter features. Strikingly, the two classes are associated with two distinct modes of nucleosome modulation. Our results suggest that the two distinct modes of nucleosome modulation are linked with the intrinsic sequence preferences for nucleosomes.

## Results

### Two promoter classes with distinct determinants of nucleosome positioning

**Figure 1:**
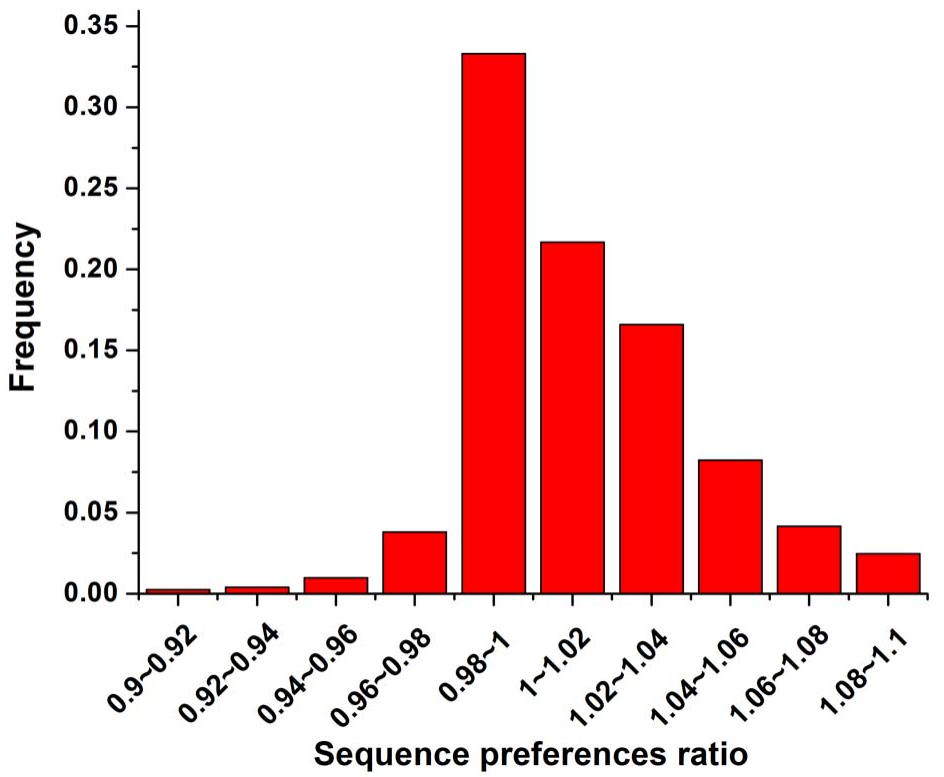
**The distribution of sequence preferences ratios for nucleosomes throughout the genome**. For each DNA sub-sequence with the length of the nucleosome, Segal et al. have predicted its sequence preferences for nucleosomes based on dinucleotide probability distributions [15]. These values were normalized, such that their values are between 0 and 1. We calculated the ratio between sequence preferences of each experimentally determined nucleosome [7] and mean preferences of linker DNA surrounding it. Distribution of all resulting ratios is shown.

**Figure 2:**
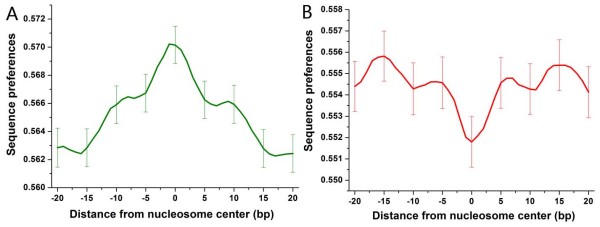
**Average sequence preferences for nucleosomes, aligned with respect to the center of the experimentally determined nucleosomes at SDN-enriched promoters (A) and SDN-less promoters (B)**. For each DNA sub-sequence with the length of the nucleosome, Segal et al. have predicted its sequence preferences for nucleosomes based on dinucleotide probability distributions [15]. These values were normalized, such that their values are between 0 and 1. The x axis corresponds to the distance between the midpoint of the sub-sequence and the center of the experimentally determined nucleosome [7]. Considering that nucleosomes were measured by DNA microarray with 4-bp resolution [7], the evident peak in the middle region of (A) indicates the sequence-dependent nucleosome positioning at SDN-enriched promoters, while the evident valley in the middle region of (B) indicates the less sequence-dependent nucleosome positioning at SDN-less promoters. Error bars were calculated by bootstrapping.

**Figure 3:**
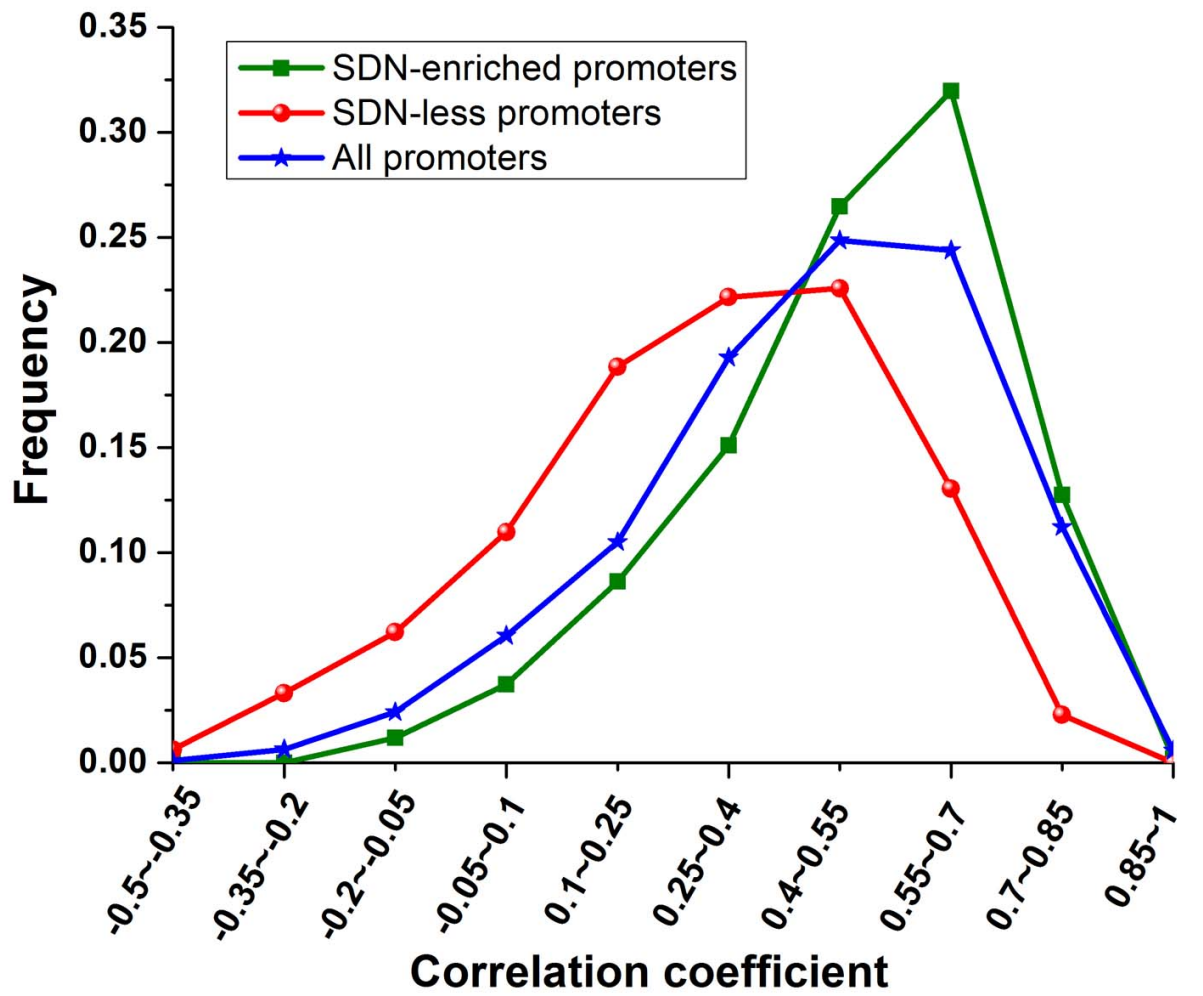
**The correlation between sequence-directed nucleosome formation potential and experimentally measured nucleosome occupancy at both promoter classes**. Lee et al. have predicted genome-wide nucleosome formation potential based on DNA features [7]. We calculated Pearson correlation coefficient between sequence-directed nucleosome formation potential and experimentally measured nucleosome occupancy [7] at each promoter. Distributions of resulting correlation coefficient values are presented for SDN-enriched promoters (green), SDN-less promoters (red) and all promoters (blue). High positive correlation indicates sequence-dependent nucleosome positioning, while low positive correlation could indicate nucleosome positioning less dependent on the intrinsic DNA sequence. SDN-enriched and SDN-less promoters showed stronger and weaker positive correlation between sequence-directed nucleosome formation potential and experimentally measured nucleosome occupancy than the rest of the promoters, respectively.

The regular spacing of certain dinucleotide sequences is known to facilitate the bending of DNA around the nucleosome [12]. Segal et al. have predicted genome-wide sequence preferences for nucleosomes in yeast based on dinucleotide probability distributions [15]. Using these data, we calculated the ratio between sequence preferences of each experimentally determined nucleosome [7] and mean preferences of linker DNA surrounding it (Figure 1), as an estimate of the dependence of nucleosome positioning on the underlying DNA sequence: High ratios indicate sequence-dependent nucleosome positioning, while low ratios could indicate nucleosome positioning less dependent on the intrinsic DNA sequence (see methods). Interestingly, the ratios of nucleosomes covering transcription start sites (TSS) and transcription termination sites (TTS) are both significantly higher than those of other nucleosomes (*P *< 10^-99 ^for TSS and *P *< 10^-94 ^for TTS, Mann-Whitney U-test). We focused on nucleosome positioning in the promoter region (1000 bp upstream of the gene in this study), and found that there were two subsets of promoters whose corresponding ratios are all greater or less than 1.01 (the median of ratios throughout the genome), respectively. This result indicates that nucleosome positioning at the two subsets of promoters is mainly or less dependent on the genomic sequence, respectively. We identified these two typical classes for subsequent analysis. Promoters were grouped into SDN (sequence-dependent nucleosomes)-enriched promoters or SDN-less promoters if their corresponding ratios were all greater or less than 1.01, respectively (510 promoters and 483 promoters respectively; Figure 2 and Additional file 1). We validated these two typical classes in another dataset [7]: SDN-enriched and SDN-less promoters showed stronger and weaker positive correlation between sequence-directed nucleosome formation potential and experimentally measured nucleosome occupancy than the rest of the promoters, respectively (Figure 3).

**Figure 4:**
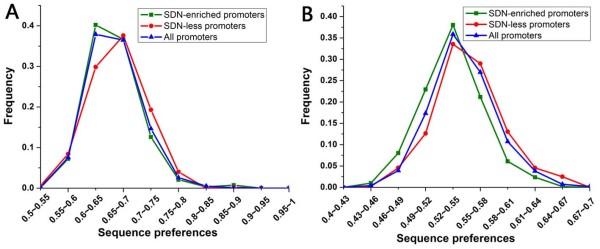
**Comparison of the intrinsic DNA sequence preferences for nucleosomes between both promoter classes**. The sequence preferences for nucleosomes were taken from Segal et al. [15]. These values were normalized, such that their values are between 0 and 1. Each sub-sequence with the length of the nucleosome was assigned a sequence preferences value for nucleosomes. For each promoter, we used the top ten maximal preferences values (A) or the mean preferences value (B) to represent its intrinsic DNA sequence preferences for nucleosomes. Distributions of preferences values are shown for SDN-enriched promoters (green), SDN-less promoters (red) and all promoters (blue).

We sought to understand why the underlying DNA sequence contributes little to nucleosome positioning at SDN-less promoters. It has been reported that the well-positioned nucleosome, which is near TSS, establishes a barrier that positions downstream nucleosomes along the coding region using the principles of statistical positioning, with little contribution from the intrinsic DNA sequence [10]. We asked whether a similar strategy is employed by SDN-less promoters. However, well-positioned nucleosomes [7] show no significant positional preference at SDN-less promoters (data not shown). We next asked whether the less sequence-dependent nucleosome positioning at SDN-less promoters is due to the low intrinsic DNA sequence preferences for nucleosomes. But our results showed that SDN-less promoters have significantly higher intrinsic sequence preferences than the rest of the promoters (*P *< 10^-28^, Mann-Whitney U-test, Figure 4). This result implies that there might be other regulatory factors that override the strong sequence preferences to position nucleosomes, resulting in less sequence-dependent nucleosome positioning at SDN-less promoters. By contrast, SDN-enriched promoters have lower intrinsic sequence preferences than the rest of the promoters (*P *< 10^-13^, Mann-Whitney U-test, Figure 4).

### Lower nucleosome occupancy can not guarantee higher transcription rate

**Figure 5:**
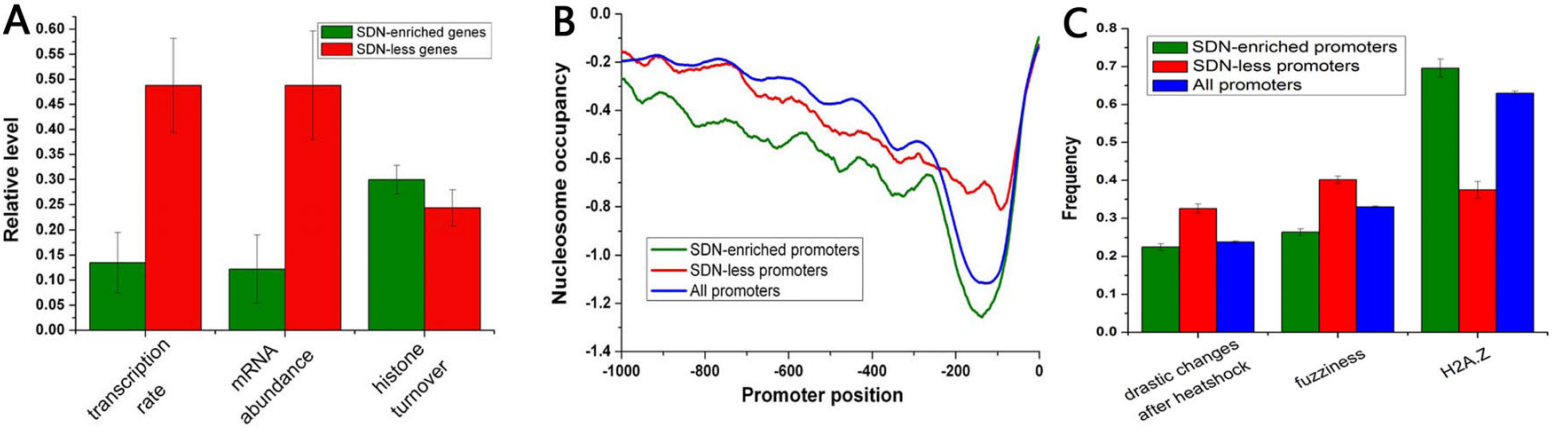
**Promoter features that distinguish the two promoter classes**. (A) Average values that correspond to transcription rate [20], gene expression level [20] and the turnover rate of H3 histone [26] are shown for SDN-enriched promoters (green) and SDN-less promoters (red). Values in each property were normalized (turnover rates were normalized among all promoters), such that their means are zero and standard deviations are one. (B) Average nucleosome profiles [7] are shown for SDN-enriched promoters (green), SDN-less promoters (red) and all promoters (blue). (C) Frequency of fuzzy nucleosomes [7] and nucleosomes with drastic positional changes (greater than 45 bp) before and after heat shock [9], as well as frequency of promoters with H2A.Z [11], is shown for SDN-enriched promoters (green), SDN-less promoters (red) and all promoters (blue). Error bars in A and C were calculated by bootstrapping.

**Figure 6:**
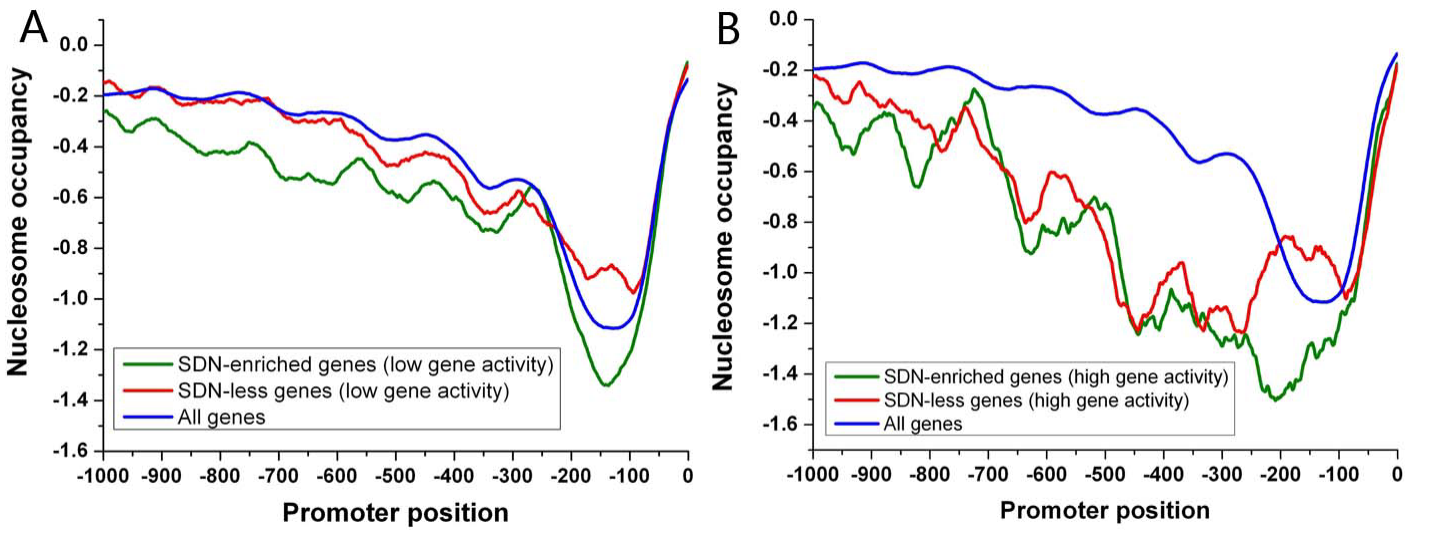
**Average nucleosome profiles are shown for subsets of SDN-enriched promoters (green), subsets of SDN-less promoters (red) and all promoters (blue)**. (A) Average nucleosome profiles [7] are shown for promoters whose downstream genes have low transcription rates [20] (normalized values less than 0) in both classes and all promoters. (B) Average nucleosome profiles are shown for promoters whose downstream genes have high transcription rates (normalized values greater than 0.5) in both classes and all promoters.

We next analyzed both classes in terms of gene activity [20], at the condition similar to that where nucleosome occupancy was measured. SDN-less genes have higher transcription rates and absolute mRNA abundance, whereas of SDN-enriched genes are comparable to genome-wide level (Figure 5A). In general, the level of nucleosome occupancy in promoter is inversely proportional to the corresponding gene transcription rate [9,21]. Strikingly, SDN-enriched promoters have significantly lower nucleosome occupancy than SDN-less promoters (Figure 5B). These results suggest that lower nucleosome occupancy can not guarantee higher transcription rate. We separately analyzed nucleosome occupancy for genes with high or low transcription rates in both classes (Figure 6). Our results demonstrate that the difference in the level of nucleosome occupancy between both classes (Figure 5B) is mainly attributable to genes with low transcription rates (Figure 6). Interestingly, SDN-less promoters lack a substantial nucleosome-free region (NFR) directly upstream of the TSS that is prevalent in most promoters [6,10]. ~500 occupied proximal-nucleosome (OPN) genes, with relatively high nucleosome occupancy close to the TSS coupled with relatively low occupancy at a more distal region in the promoter, show higher mRNA expression levels [22]. There is only a small overlap (~13%) in membership between SDN-less and OPN genes, implying that they capture distinct nucleosomal features.

**Figure 7:**
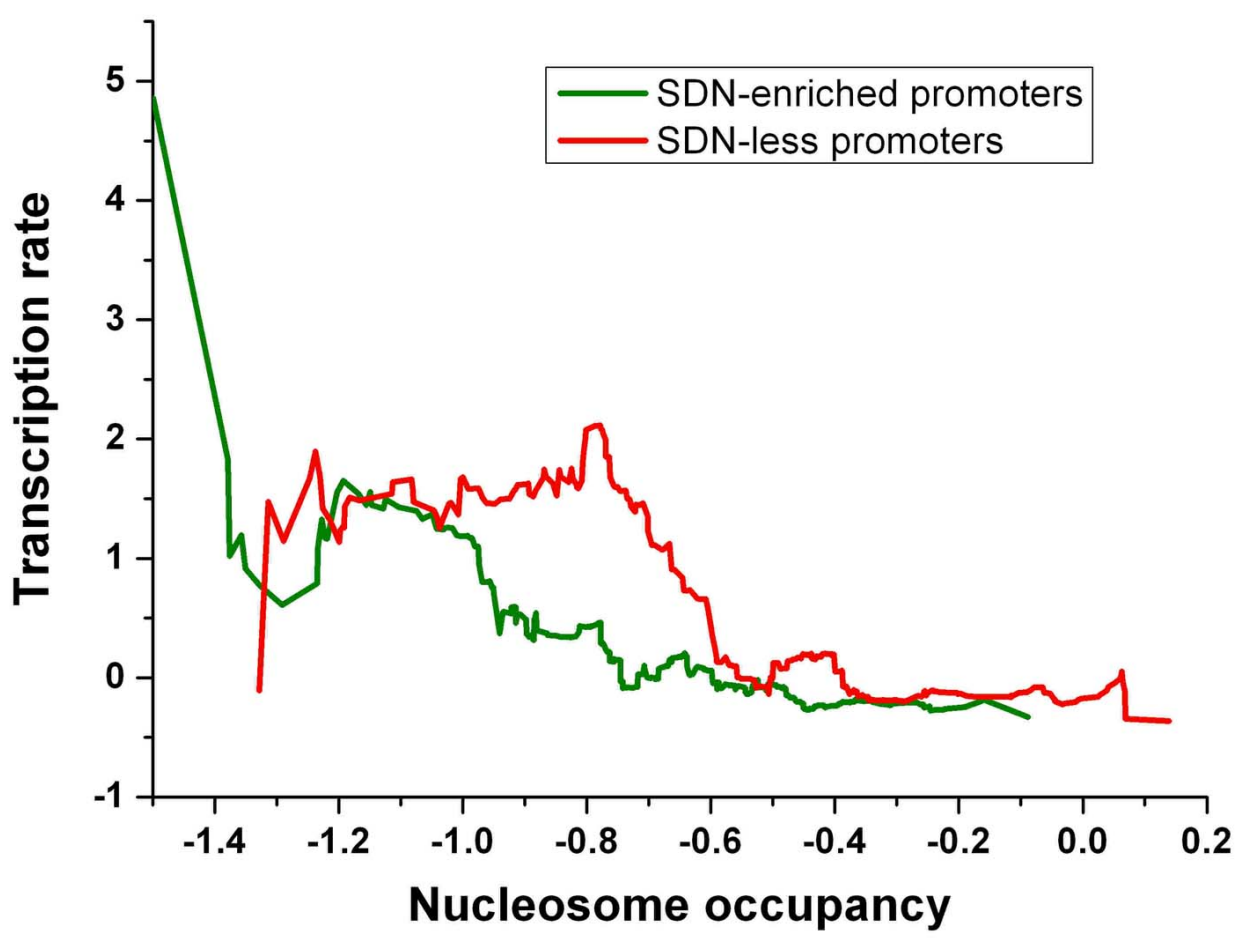
**Correlation between nucleosome occupancy and transcription rate for SDN-enriched promoters (green) and SDN-less promoters (red)**. Genes were ordered by their average occupancy [7] at the promoter, and the normalized transcription rate data [20] were smoothed over a sliding window with window size of 50 genes.

**Figure 8:**
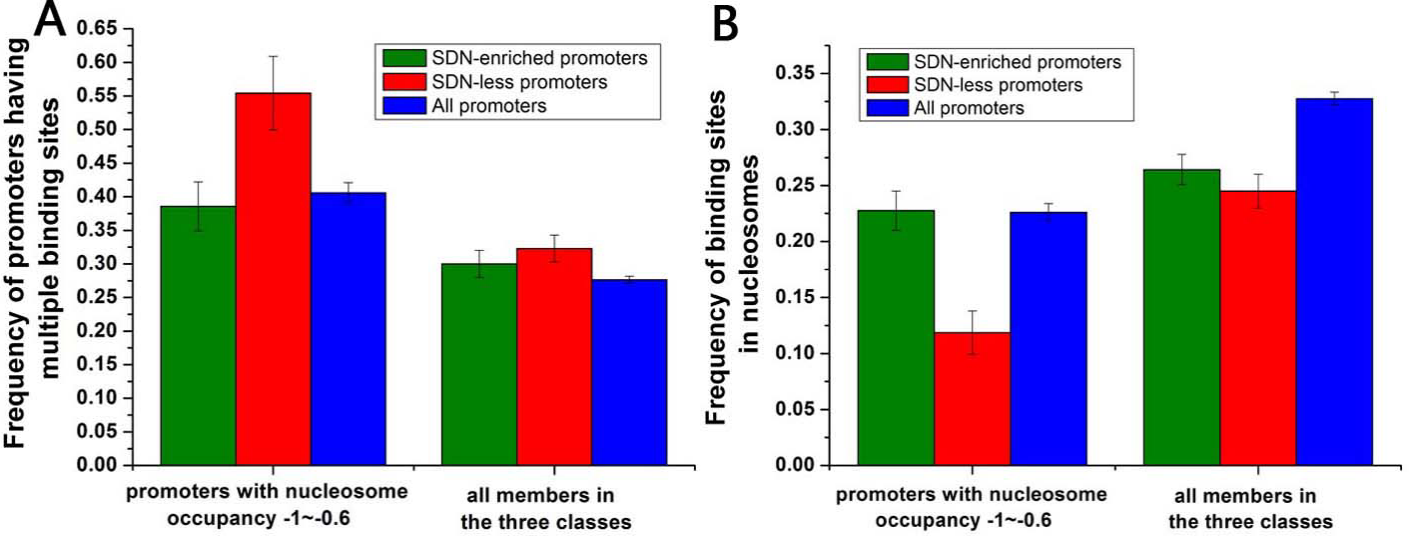
**Transcription factor binding sites at both promoter classes**. (A) Frequency of promoters with multiple (greater than 1) transcription factor binding sites [23] is shown for SDN-enriched promoters (green), SDN-less promoters (red), all promoters (blue), and subsets of these promoter classes whose average nucleosome occupancy is between -1 and -0.6. (B) Frequency of transcription factor binding sites [23] localized in nucleosome [7] is shown for SDN-enriched promoters (green), SDN-less promoters (red), all promoters (blue), and subsets of these promoter classes whose average nucleosome occupancy is between -1 and -0.6. Error bars in A and B were calculated by bootstrapping.

We further investigated into the relationship between nucleosome occupancy and transcription rate. Transcription rates tend to decrease with increasing nucleosome occupancy at promoters for SDN-enriched genes, while this trend is relatively weak for SDN-less genes (Figure 7). For promoters with similar nucleosome occupancy between -1 and -0.6, SDN-less genes have significantly higher transcription rates than SDN-enriched genes (Figure 7). We restricted the analysis to promoters whose nucleosome occupancy is in this interval. We asked whether transcription factor (TF) binding information contributes to the significant difference in transcription rate. Indeed, SDN-less promoters are enriched with transcription factor binding sites [23] compared with SDN-enriched promoters (Figure 8A). Moreover, binding sites are highly localized in linker DNA [7] at SDN-less promoters (Figure 8B). These significantly discriminative characteristics disappeared when we included all members in both classes (Figure 8).

### Two distinct modes of nucleosome modulation

**Figure 9:**
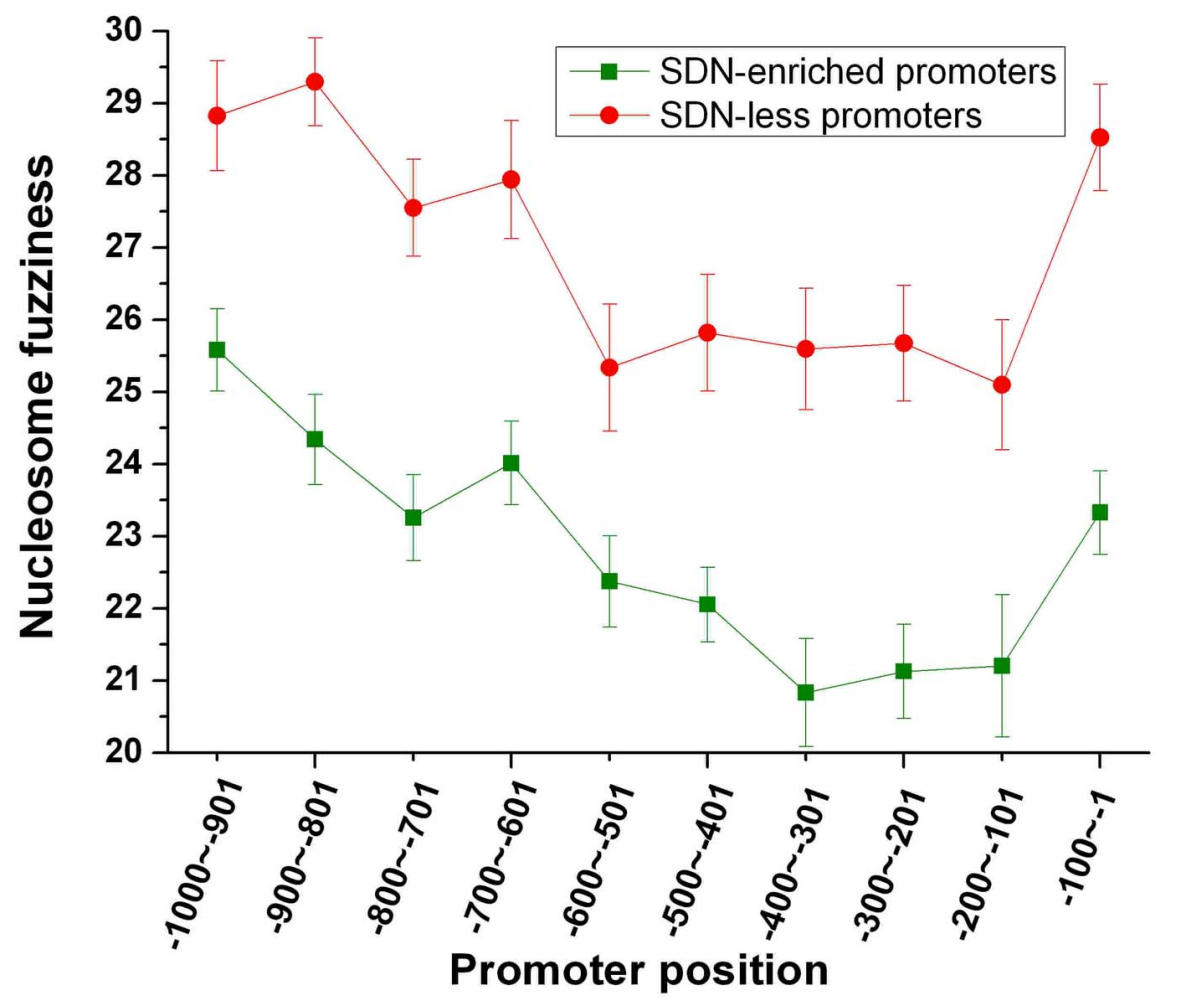
**Nucleosome fuzziness at both promoter classes**. Average nucleosome fuzziness [10] along the promoter is shown for SDN-enriched promoters (green) and SDN-less promoters (red). Error bars were calculated by bootstrapping.

**Figure 10:**
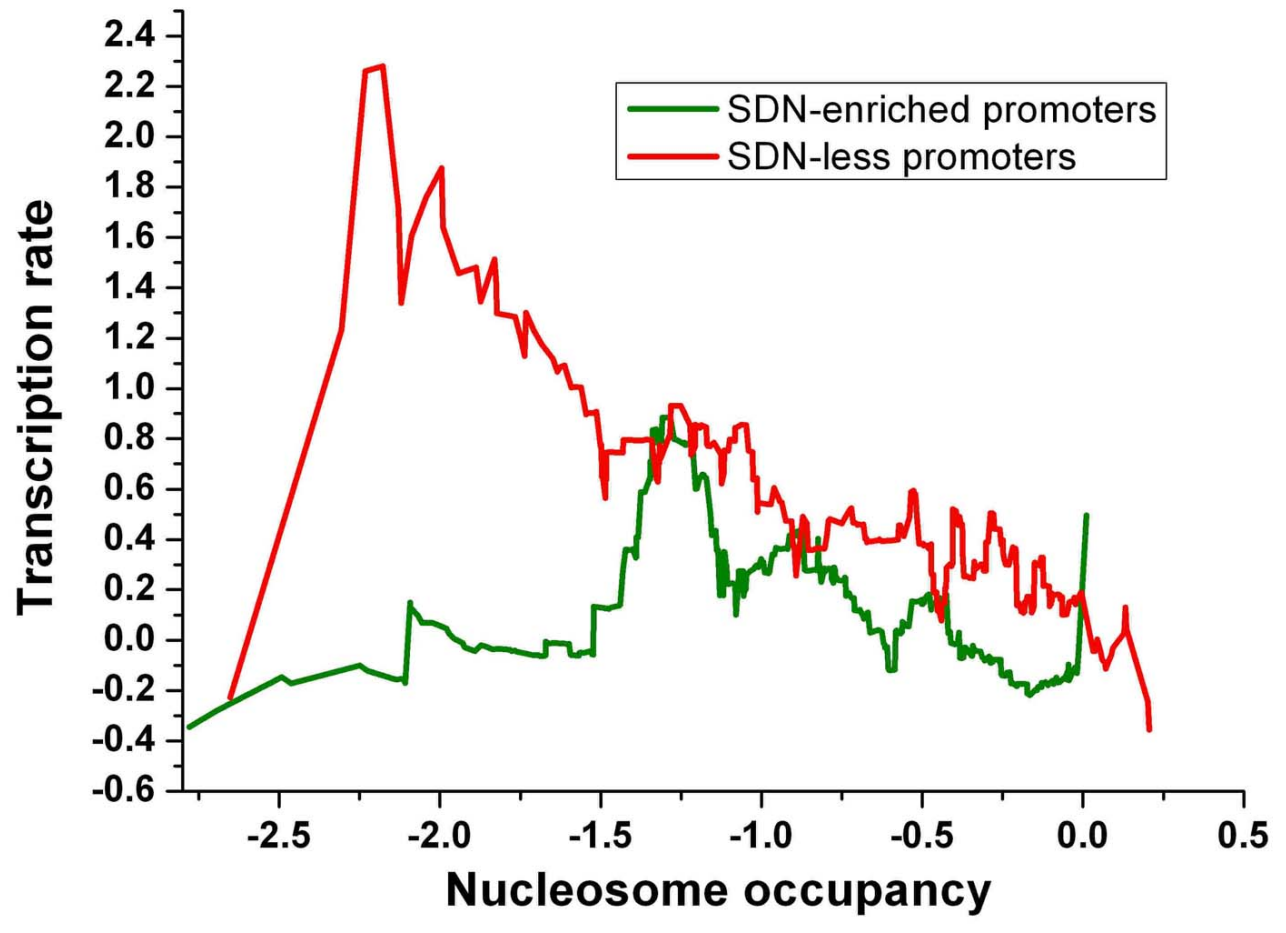
**Correlation between nucleosome occupancy and transcription rate for SDN-enriched promoters (green) and SDN-less promoters (red)**. Genes were ordered by their average occupancy [7] within the 150 bp upstream of the gene, and the normalized transcription rate data [20] were smoothed over a sliding window with window size of 50 genes.

We examined whether there is significant difference in nucleosome properties between the two classes. SDN-less promoters are enriched with delocalized (fuzzy) nucleosomes [7] (Figure 5C). Analysis of nucleosome fuzziness measured in another study [10] reproduces a similar observation (Figure 9). These results suggest that nucleosome positioning at SDN-less promoters is more dynamic. Nucleosomes surrounding TSS at SDN-less promoters show high fuzziness (Figure 9). Moreover, for promoters with similar nucleosome occupancy [7] within the 150 bp upstream of the gene, SDN-less genes have significantly higher transcription rates [20] than SDN-enriched genes (Figure 10). One possible explanation is that high RNA polymerase II occupancy, which is generally associated with high rate of transcription [24], should exclude nucleosomes from more distinct positions (more nucleosome fuzziness) in the measured ensemble. Two H2A.Z nucleosomes flank NFR at most promoters in yeast [25]. As expected, SDN-less promoters are depleted of H2A.Z nucleosomes [11] (Figure 5C), By contrast, SDN-enriched promoters are relatively enriched with H2A.Z nucleosomes and are depleted of 'fuzzy' nucleosomes (Figure 5C). Comparison of nucleosome positions between normal and heat-shock conditions [9] showed that SDN-less promoters are enriched with nucleosomes with drastic positional changes (Figure 5C). However, the changes in overall nucleosome occupancy (before and after heat shock) at the two classes of promoters are both comparable to genome-wide level (data not shown).

**Figure 11:**
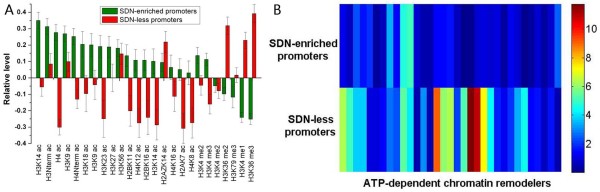
**Two distinct modes of nucleosome modulation for the two promoter classes**. (A) Average values that quantify the levels of histone modification [33,34] are shown for SDN-enriched promoters (green), SDN-less promoters (red). Values in each histone modification were normalized, such that their means are zero and standard deviations are one. Ac represents acetylation, while me indicates methylation (e.g. me2 indicates dimethylation). Error bars were calculated by bootstrapping. (B) A heatmap of ATP-dependent chromatin remodeling. Rows represent both promoter classes, and columns represent ATP-dependent chromatin remodelers (See additional file 1 for the corresponding list). Colors indicate K-S -log_10_*P *value, which provide a measure of the discrepancy in expression between SDN-enriched or SDN-less promoters and the rest of the promoters when ATP-dependent chromatin remodeler gene is mutated. Higher values indicate higher discrepancy.

Both SDN-enriched and SDN-less promoters exhibit higher turnover rates of histone H3 [26] (Figure 5A). In general, there are two main ways in which cells modulate nucleosomes: Histone modification and ATP-dependent chromatin remodelling [27,28]. Importantly, SDN-enriched promoters are characterized by hyperacetylation, while SDN-less promoters tend to be hypoacetylation (Figure 11A). Different from other known modifications, acetylation can neutralize the positive charge of the lysine. As a result, acetylated histone tails are thought to associate more loosely with nucleosomal DNA than unmodified and methylated histone tails [29]. We used a compendium of gene expression experiments in which various ATP-dependent chromatin remodelers were deleted or mutated [30] (Additional file 1). If the remodeler regulates a subset of genes, its deletion should cause a differential change in expression. We used the Kolmogorov-Smirnov (K-S) statistical test to evaluate the difference in the distribution of gene expression values between a subset of genes and the rest of the genes. SDN-less promoters are highly regulated by ATP-dependent chromatin remodelers, while SDN-enriched promoters show very low sensitivity to disruption of these remodelers (Figure 11B).

## Discussion

Previous studies have shown that the genomic sequence itself can explain ~50% of the in vivo nucleosome organization in yeast [15,16]. These results indicate that some nucleosome positions are mainly determined by the genomic sequence, while other nucleosome positions are less determined by DNA. However, it is still unclear whether these two nucleosome classes could possess different properties and might influence the properties of their located genes. To address this issue, we identified two typical promoter classes whose nucleosome positions are mainly or less dependent on the underlying DNA sequence, respectively. Importantly, these two promoter classes can be distinguished by multiple promoter features and modes of nucleosome modulation.

SDN-enriched promoters have lower nucleosome occupancy and lower transcription rates compared with SDN-less promoters (Figure 5). This is a very interesting observation since the level of nucleosome occupancy in promoter is generally inversely proportional to the corresponding gene transcription rate [21]. We suggest that nucleosome occupancy at promoter establishes the context in which transcription operates, while transcription apparatus specifies the rate of transcription. It is well accepted that TFs bind their targets in a dynamic manner [31]. SDN-less promoters are enriched with fuzzy nucleosomes compared with SDN-enriched promoters (Figure 5 and figure 9), suggesting that nucleosome positioning at SDN-less promoters is more dynamic. This nucleosomal dynamics may guide transcription factors to their targets sites in a dynamic manner, although SDN-less promoters have higher nucleosome occupancy than SDN-enriched promoters (Figure 5).

Histone modification has been thought to work in concert with ATP-dependent chromatin remodelling to regulate nucleosome mobility [27,28], but the two promoter classes employed distinct modes of nucleosome modulation: Hyperacetylation for SDN-enriched promoters and ATP-dependent chromatin remodelling for SDN-less promoters. These observations along with high intrinsic sequence preferences for nucleosomes at SDN-less promoters (Figure 4) indicate that they need to use more energy in the form of ATP hydrolysis to override the underlying DNA sequence to regulate nucleosome mobility. ATP-dependent chromatin remodeling often alters nucleosome position [3], probably resulting in less sequence-dependent nucleosome positioning and high nucleosome fuzziness at SDN-less promoters. On the other hand, SDN-enriched promoters need not use much energy (mainly in the form of acetylation) to loosen histone-DNA association partly due to their relatively low intrinsic sequence preferences for nucleosomes (Figure 4). Acetylation is unlikely to transfer nucleosome position directly [29], which may maintain the contribution of the underlying DNA sequence to nucleosome positioning at SDN-enriched promoters.

## Conclusion

We identified two typical promoter classes according to the degree of dependence of nucleosome positioning on the underlying DNA sequence. Their difference in contribution of the genomic sequence to nucleosome positioning appears to be a consequence of their distinct modes of nucleosome modulation: hyperacetylation for SDN-enriched promoters and ATP-dependent chromatin remodelling for SDN-less promoters. The adoption of distinct modes of nucleosome modulation by the two promoter classes may be attributable to their difference in the intrinsic sequence preferences for nucleosomes (see discussion), and may result in different promoter features. ATP-dependent chromatin remodeling often changes nucleosome position, probably resulting in high nucleosomal dynamics (fuzziness) at SDN-less promoters, while acetylation is unlikely to transfer nucleosome position directly, which leads to the well-positioned nucleosomes (low nucleosome fuzziness) at SDN-enriched promoters. Our findings should facilitate the understanding of how in vivo nucleosome positioning is coordinated.

## Methods

### The identification of two typical promoter classes

For each DNA sub-sequence with the length of the nucleosome in yeast, Segal et al. have predicted its sequence preferences for nucleosomes based on dinucleotide probability distributions [15]. The March 2008 version of dataset was downloaded from Dr. Segal's website. The data were normalized, such that their values are between 0 and 1. Lee et al. have experimentally identified genome-wide nucleosome positions in yeast [7]. We first mapped the sequence preferences value to each experimentally determined nucleosome, and next calculated the mean preferences for all nucleosomal-long sequences (except the one bound by the nucleosome) that are between the right end of its previous adjacent nucleosome and the left end of its next adjacent nucleosome. We used this mean value to represent sequence preferences of linker DNA surrounding the nucleosome. For each experimentally determined nucleosome, we calculated the ratio between its sequence preferences and mean preferences of linker DNA adjacent to it (Figure 1), as an estimate of the dependence of its positioning on the underlying DNA sequence: High ratios indicate sequence-dependent nucleosome positioning, while low ratios could indicate nucleosome positioning less dependent on the intrinsic DNA sequence. Previous studies have shown that the genomic sequence itself can explain ~50% of the in vivo nucleosome organization in yeast [15,16]. We focused on nucleosome positioning in the promoter region (1000 bp upstream of the gene in this study) [32], and found that there were two subset of promoters whose corresponding ratios are all greater or less than 1.01 (the median of ratios throughout the genome), respectively. This result indicates that nucleosome positioning at the two subsets of promoters is mainly or less dependent on the genomic sequence, respectively. We identified these two typical classes for subsequent analysis. Promoters were grouped into SDN (sequence-dependent nucleosomes)-enriched promoters or SDN-less promoters if their corresponding ratios were all greater or less than 1.01, respectively (510 promoters and 483 promoters respectively; Figure 2 and Additional file 1).

To validate the two typical promoter classes, we examined the ratio between sequence preferences of a nucleosome and maximum preferences of linker DNA surrounding it. SDN-enriched and SDN-less promoters had significantly higher and lower ratios compared with genome-wide level, respectively (*P *< 10^-7 ^for SDN-enriched promoters and *P *< 10^-10 ^for SDN-less promoters, Mann-Whitney U-test). We also used another dataset of nucleosome formation potential predicted from DNA features [7], and calculated its correlation with experimentally determined nucleosome occupancy [7] at each promoter (Figure 3).

### Nucleosomal data

The positions of well-positioned and delocalized (fuzzy) nucleosomes were taken from Lee et al. [7]. For the two promoter classes, we calculated the percentage of fuzzy nucleosomes, respectively. For SDN-less promoters, we calculated the positional distribution of well-positioned nucleosomes. The nucleosome fuzziness data was taken from Mavrich et al. [10]. For the two promoter classes, we calculated the average fuzziness along the promoter, respectively. H2A.Z nucleosomes were taken from Albert et al. [11]. To avoid confusion, we restricted the analysis to the 10% most scored H2A.Z nucleosomes in the literature. For both promoter classes, we calculated the percentage of promoters containing H2A.Z nucleosomes, respectively. Nucleosome occupancy in normal and heat-shock conditions were taken from Shivaswamy et al. [9]. ~65% of all nucleosomes throughout the genome in normal condition were within 30 bp of their positions in heat-shock condition. For both promoter classes, we calculated the percentage of nucleosomes with drastic positional changes (greater than 45 bp) before and after heat shock. We also calculated the changes in the number of nucleosomes in each promoter between the two conditions. Turnover rates of histone H3 were taken from Dion et al. [26], which were normalized among all promoters in that turnover rates at promoters are higher than those in coding region [26], such that their means are zero and standard deviations are one. The data of sequence preferences for nucleosomes were taken from Segal et al. [15], which were normalized, such that their values are between 0 and 1. We compared the intrinsic DNA sequence preferences between SDN-less promoters (or SDN-enriched promoters) and the rest of the promoters by considering the top ten maximal preferences values or the mean value at each promoter.

### Gene activity and transcription factor binding sites

The transcription rates and mRNA abundance were taken from Holstege et al. [20], which were normalized, such that their means are zero and standard deviations are one. Transcription factor binding sites were taken from Harbison et al. [23], which includes the binding sites of 203 TFs at promoters in YPD medium. A *P *value cutoff of 0.001 was used to define the set of genes bound by a particular TF. For every subset of promoters considered in the main text, we calculated the percentage of promoters having multiple transcription factor binding sites, and the percentage of binding sites localized in nucleosome [7].

### Histone modification and ATP-dependent chromatin remodeling

Histone modification data were taken from ChromationDB [33], a database of genome-wide histone modification patterns for Saccharomyces cerevisiae. We added the histone modification data from Pokholok et al. [34], resulting in a total of 25 histone modifications. Values have been normalized in the literature. For each promoter, we considered the average level for each histone modification. We used a compendium of gene expression experiments in which various ATP-dependent chromatin remodelers were deleted or mutated [30] (Additional file 1). If the remodeler regulates a subset of genes, its deletion should cause a differential change in expression. We used the Kolmogorov-Smirnov (K-S) statistical test to evaluate the difference in the distribution of gene expression values between a subset of genes and the rest of the genes. K-S -log_10_*P *value, which provide a measure of the discrepancy in expression between SDN-enriched or SDN-less promoters and the rest of the promoters when ATP-dependent chromatin remodeler gene is mutated. Higher values indicate higher discrepancy.

## Authors' contributions

ZD and XD designed the study, analyzed the results and drafted the manuscript, and ZD also implemented the algorithms, carried out the experiments. QX, JF, YD, JW and CH participated in the analysis and discussion. All authors read and approved the final manuscript.

## Supplementary Material

Additional file 1 **Table S1 and Table S2. The lists of both promoter classes and ATP-dependent chromatin remodelers**. Table S1 lists ORF names for the 510 SDN (sequence-dependent nucleosomes)-enriched genes and 483 SDN-less genes. Table S2 lists ATP-dependent chromatin remodelers that correspond to the ordered columns in Figure 11B. Click here for file

## References

[B1] Richmond TJ, Davey CA (2003). The structure of DNA in the nucleosome core. Nature.

[B2] Luger K, Hansen JC (2005). Nucleosome and chromatin fiber dynamics. Curr Opin Struc Biol.

[B3] Flaus A, Owen-Hughes T (2001). Mechanisms for ATP-dependent chromatin remodelling. Curr Opin Genet Dev.

[B4] Kouzarides T (2007). Chromatin modifications and their function. Cell.

[B5] Henikoff S, Ahmad K (2005). Assembly of variant histones into chromatin. Annu Rev Cell Dev Biol.

[B6] Yuan G, Liu Y, Dion MF, Slack MD, Wu LF, Altschuler SJ, Rando OJ (2005). Genome-scale identification of nucleosome positions in S. cerevisiae. Science.

[B7] Lee W, Tillo D, Bray N, Morse RH, Davis RW, Hughes TR, Nislow C (2007). A high-resolution atlas of nucleosome occupancy in yeast. Nat Genet.

[B8] Whitehouse I, Rando OJ, Delrow J, Tsukiyama T (2007). Chromatin remodelling at promoters suppresses antisense transcription. Nature.

[B9] Shivaswamy S, Bhinge A, Zhao Y, Jones S, Hirst M, Iyer VR (2008). Dynamic remodeling of individual nucleosomes across a eukaryotic genome in response to transcriptional perturbation. PLoS Biol.

[B10] Mavrich TN, Ioshikhes IP, Venters BJ, Jiang C, Tomsho LP, Qi J, Schuster SC, Albert I, Pugh BF (2008). A barrier nucleosome model for statistical positioning of nucleosomes throughout the yeast genome. Genome Res.

[B11] Albert I, Mavrich TN, Tomsho LP, Qi J, Zanton SJ, Schuster SC, Pugh BF (2007). Translational and rotational settings of H2A.Z nucleosomes across the Saccharomyces cerevisiae genome. Nature.

[B12] Widom J (2001). Role of DNA sequence in nucleosome stability and dynamics. Q Rev Biophys.

[B13] Thastrom A, Lowary PT, Widlund HR, Cao H, Kubista M, Widom J (1999). Sequence motifs and free energies of selected natural and non-natural nucleosome positioning DNA sequences. J Mol Biol.

[B14] Yuan GC, Liu JS (2008). Genomic sequence is highly predictive of local nucleosome depletion. PLoS Comput Biol.

[B15] Segal E, Fondufe-Mittendorf Y, Chen L, Thastrom A, Field Y, Moore IK, Wang JZ, Widom J (2006). A genomic code for nucleosome positioning. Nature.

[B16] Pechham HE, Thurman RE, Fu Y, Stamatoyannopoulos JA, Noble WS, Struhl K, Weng Z (2007). Nucleosome positioning signals in genomic DNA. Genome Res.

[B17] Ioshikhes IP, Albert I, Zanton SJ, Pugh BF (2006). Nucleosome positions predicted through comparative genomics. Nat Genet.

[B18] Miele V, Vaillant C, Aubenton-Carafa Y, Thermes C, Grange T (2008). DNA physical properties determine nucleosome occupancy from yeast to fly. Nucleic Acids Res.

[B19] Whitehouse I, Tsukiyama T (2006). Antagonistic forces that position nucleosomes in vivo. Nature Struct Mol Biol.

[B20] Holstege FCP, Jennings EG, Wyrich JJ, Lee TI, Hengartner CJ, Green MR, Golub TR, Lander ES, Young RA (1998). Dissecting the regulatory circuitry of a eukaryotic genome. Cell.

[B21] Lee CK, Shibata Y, Rao B, Strahl BD, Lieb JD (2004). Evidence for nucleosome depletion at active regulatory regions genome-wide. Nat Genet.

[B22] Tirosh I, Barkai N (2008). Two strategies for gene regulation by promoter nucleosomes. Genome Res.

[B23] Harbison CT, Gordon DB, Lee TI, Rinaldi NJ, Macisaac KD, Danford TW, Hannett NM, Tagne J, Reynolds DB, Yoo J, Jennings EG, Zeitlinger J, Pokholok DK, Kellis M, Rolfe PA, Takusagawa KT, Lander ES, Gifford DK, Fraenkel E, Young RA (2004). Transcriptional regulatory code of a eukaryotic genome. Nature.

[B24] Steinmetz EJ, Warren CL, Nuehner JN, Panbehi B, Ansari AZ, Brow DA (2006). Genome-wide distribution of yeast RNA polymerase II and its control by sen1 helicase. Mol Cell.

[B25] Raisner RM, Hartley PD, Meneghini MD, Bao MZ, Liu CL, Schreiber SL, Rando OJ, Madhani HD (2005). Histone variant H2A.Z marks the 5' Ends of both active and inactive genes in euchromatin. Cell.

[B26] Dion MF, Kaplan T, Kim M, Buratowski S, Friedman N, Rando OJ (2007). Dynamics of replication-independent histone turnover in budding yeast. Science.

[B27] Cosgrove MS, Boeke JD, Wolberger C (2004). Regulated nucleosome mobility and the histone code. Nat Struct Mol Biol.

[B28] Narlikar GJ, Fan HY, Kingston RE (2002). Cooperation between complexes that regulate chromatin structure and transcription. Cell.

[B29] Shahbazian MD, Grunstein M (2007). Functions of site-specific histone acetylation and deacetylation. Annu Rev Biochem.

[B30] Steinfeld I, Shamir R, Kupiec M (2007). A genome-wide analysis in Saccharomyces cerevisiae demonstrates the influence of chromatin modifiers on transcription. Nat Genet.

[B31] Mellor J (2005). The dynamics of chromatin remodeling at promoters. Mol Cell.

[B32] Saccharomyces Genome Database. http://www.yeastgenome.org.

[B33] O'Connor TR, Wyrick JJ (2007). ChromatinDB: a database of genome-wide histone modification patterns for Saccharomyces cerevisiae. Nucleic Acids Res.

[B34] Pokholok DK, Harbison CT, Levine S, Cole M, Hannett NM, Lee TI, Bell GW, Walker K, Rolfe PA, Herbolsheimer E, Zeitlinger J, Lewitter F, Gifford DK, Young RA (2005). Genome-wide map of nucleosome acetylation and methylation in yeast. Cell.

