# Supplementary material for: Two distinct modes of nucleosome modulation associated with different degrees of dependence of nucleosome positioning on the underlying DNA sequence
Source: BMC Genomics. 2009 Jan 10;10:15. doi: 10.1186/1471-2164-10-15 (PMC2631480; doi:10.1186/1471-2164-10-15)
Supplement: Additional file 1 — Table S1 and Table S2. The lists of both promoter classes and ATP-dependent chromatin remodelers. Table S1 lists ORF names for the 510 SDN (sequence-dependent nucleosomes)-enriched genes and 483 SDN-less genes. Table S2 lists ATP-dependent chromatin remodelers that correspond to the ordered columns in Figure 11B. [file 1471-2164-10-15-S1.pdf]

Table S1 ORF names for the 510 SDN (sequence-dependent nucleosomes)-enriched genes and 483 SDN-less genes

SDN-enriched genes	SDN-less genes
YAL054C	YAL065C
YAL053W	YAL064W
YAL025C	YAL038W
YAL016W	YAR009C
YAL003W	YAR028W
YAR061W	YBL113C
YAR073W	YBL112C
YBL105C	YBL111C
YBL091C	YBL109W
YBL089W	YBL106C
YBL088C	YBL093C
YBL086C	YBL092W
YBL059C-A	YBL078C
YBL058W	YBL074C
YBL054W	YBL071W-A
YBL043W	YBL048W
YBL035C	YBL042C
YBL026W	YBL041W
YBL003C	YBL029C-A
YBR033W	YBL028C
YBR054W	YBR006W
YBR059C	YBR009C
YBR060C	YBR012C
YBR066C	YBR017C
YBR074W	YBR048W
YBR085C-A	YBR053C
YBR086C	YBR068C
YBR122C	YBR072W
YBR127C	YBR077C
YBR158W	YBR078W
YBR162C	YBR081C
YBR163W	YBR085W
YBR180W	YBR101C
YBR253W	YBR118W
YBR255W	YBR121C-A
YBR255C-A	YBR128C
YBR265W	YBR153W
YBR271W	YBR156C
YBR291C	YBR183W
YBR293W	YBR234C

YBR295W	YBR243C
YCL054W	YBR296C
YCL050C	YCL069W
YCL040W	YCL063W
YCL002C	YCL028W
YCL001W-A	YCL027W
YCL001W-B	YCL021W-A
YCR030C	YCR024C-A
YCR039C	YCR034W
YCR042C	YCR049C
YCR044C	YCR066W
YCR045W-A	YCR102C
YCR073W-A	YCR106W
YCR084C	YDL210W
YCR086W	YDL186W
YCR090C	YDL177C
YCR091W	YDL152W
YDL240W	YDL136W
YDL205C	YDL080C
YDL204W	YDL075W
YDL195W	YDL062W
YDL179W	YDL045C
YDL171C	YDL039C
YDL170W	YDL036C
YDL158C	YDL023C
YDL156W	YDL019C
YDL138W	YDL018C
YDL130W	YDL017W
YDL081C	YDR007W
YDL068W	YDR011W
YDL069C	YDR019C
YDL066W	YDR021W
YDL055C	YDR033W
YDL048C	YDR043C
YDL047W	YDR044W
YDL022C-A	YDR046C
YDR008C	YDR047W
YDR009W	YDR050C
YDR028C	YDR061W
YDR035W	YDR072C
snR47	YDR114C
YDR054C	YDR129C
YDR055W	YDR133C
YDR088C	YDR134C

YDR096W	YDR153C
YDR106W	YDR156W
YDR107C	YDR171W
YDR109C	YDR187C
YDR116C	YDR189W
YDR125C	YDR209C
YDR135C	YDR249C
YDR144C	YDR270W
YDR152W	YDR269C
YDR183W	YDR280W
YDR199W	YDR302W
YDR201W	YDR323C
YDR213W	YDR345C
YDR231C	YDR355C
YDR246W	YDR362C
YDR253C	YDR363W
YDR257C	YDR363W-A
YDR259C	YDR365C
YDR281C	YDR379C-A
YDR291W	YDR382W
YDR306C	YDR384C
YDR307W	YDR393W
YDR320W-B	YDR394W
YDR321W	YDR406W-A
YDR328C	YDR441C
YDR354C-A	YDR442W
YDR356W	YDR449C
YDR370C	YDR450W
YDR402C	YDR452W
YDR403W	YDR502C
YDR406W	YDR509W
YDR408C	YDR526C
YDR410C	YEL077W-A
YDR419W	YEL073C
YDR420W	YEL057C
YDR428C	YEL047C
YDR458C	YEL025C
YDR461C-A	YEL024W
YDR463W	YEL010W
YDR470C	YEL008C-A
YDR479C	YEL007W
YDR500C	YER004W
YDR508C	YER014W
YDR510W	YER014C-A

YDR511W	YER029C
YDR524C	YER055C
YDR525W	YER062C
YDR525W-A	YER063W
YEL054C	YER064C
YEL053C	YER065C
YEL050W-A	YER096W
YEL048C	YER101C
YEL046C	YER109C
YEL045C	YER117W
YEL044W	YER131W
YEL043W	YER136W
YEL036C	YER137C-A
YEL018C-A	YER138C
YEL017C-A	YER138W-A
YEL017W	YER145C-A
YEL016C	YER147C-A
YER002W	YER190C-A
YER018C	YER190C-B
YER031C	YFL066C
YER049W	YFL059W
YER056C	YFR009W-A
YER070W	YFR017C
YER083C	YFR052C-A
YER086W	YGL262W
YER089C	YGL253W
YER100W	YGL235W
YER124C	YGL208W
YER125W	YGL191W
YER172C	YGL189C
YER180C-A	YGL188C-A
YER182W	YGL172W
YFL055W	YGL135W
YFL034C-B	YGL062W
YFL034W	YGL038C
YFL026W	YGL027C
YFR012W-A	YGL014C-A
YFR013W	YGL008C
YFR025C	YGR004W
YFR031C-A	YGR022C
YFR034C	YGR035C
YFR034W-A	YGR041W
YFR036W	YGR047C
YFR054C	YGR055W

YGL255W	YGR060W
YGL237C	YGR085C
YGL219C	YGR090W
YGL190C	YGR094W
YGL175C	YGR100W
YGL158W	YGR115C
YGL144C	YGR117C
YGL141W	YGR121C
YGL137W	YGR121W-A
YGL128C	YGR122W
YGL126W	YGR148C
YGL124C	YGR171C
YGL123W	YGR182C
YGL076C	YGR190C
YGL065C	YGR201C
YGL060W	YGR203W
YGL035C	YGR213C
YGL020C	YGR214W
YGL018C	YGR224W
YGL017W	YGR244C
YGL007C-A	YGR251W
YGR058W	YGR259C
YGR062C	YGR279C
YGR068C	YGR287C
YGR098C	YGR288W
YGR110W	YGR296C-A
YGR118W	YGR296C-B
YGR138C	YHL046W-A
YGR159C	YHL042W
YGR167W	YHL037C
YGR181W	YHL029C
YGR187C	YHL028W
YGR189C	YHR007C
YGR204W	YHR021W-A
YGR221C	YHR025W
YGR222W	YHR043C
YGR229C	YHR050W
YGR230W	YHR063C
YGR234W	YHR072W-A
YGR240C	YHR094C
YGR254W	YHR097C
YGR282C	YHR139C
YHL044W	YHR141C
YHL018W	YHR171W

YHL017W	YHR178W
YHL013C	YHR183W
YHL007C	YHR182C-A
YHL004W	YHR204W
YHL003C	YHR211W
YHR001W-A	YHR217C
YHR002W	YHR218W
YHR021C	YHR218W-A
YHR072W	YHR219W
YHR073W-A	YHR219C-A
YHR073C-B	YIL171W-A
YHR077C	YIL119C
YHR088W	YIL107C
YHR098C	YIL100W
YHR145C	YIL098C
YHR152W	YIL069C
YHR155W	YIL066W-A
YHR156C	YIL056W
YHR174W	YIL055C
YHR188C	YIL051C
YHR190W	YIL050W
YIL145C	YIL048W
YIL137C	YIL037C
YIL136W	YIL035C
YIL130W	YIL020C-A
YIL102C	YIL019W
YIL075C	YIL018W
YIL057C	YIL013C
YIL029C	YIL011W
YIL026C	YIL002C
YIL016W	YIR019C
YIL008W	YJL220W
YIR001C	YJL203W
YIR016W	YJL201W
YIR017C	YJL195C
YIR027C	YJL192C
YIR028W	YJL143W
YJL196C	YJL142C
YJL189W	YJL135W
YJL187C	YJL105W
YJL177W	YJL089W
YJL168C	YJL077W-B
YJL158C	YJL032W
YJL121C	YJL020W-A

YJL119C	YJR004C
YJL117W	YJR005W
YJL098W	YJR010W
YJL083W	YJR010C-A
YJL064W	YJR042W
YJL063C	YJR057W
YJL062W	YJR121W
YJL057C	YJR128W
YJL052W	YJR148W
YJL038C	YJR149W
YJL037W	YKL213C
YJL020C	YKL161C
YJL007C	YKL152C
YJR003C	YKL142W
YJR044C	YKL141W
YJR047C	YKL124W
YJR048W	YKL067W
YJR055W	YKL034W
YJR083C	YKL011C
YJR100C	YKL006W
YJR109C	YKR015C
YJR111C	YKR016W
YJR112W-A	YKR035C
YJR127C	YKR072C
YJR135C	YKR074W
YJR138W	YKR093W
YJR147W	YKR104W
YKL217W	YLL066W-A
YKL209C	YLL062C
YKL194C	YLL048C
YKL175W	YLL047W
YKL165C	YLL039C
YKL157W	YLL028W
YKL145W	YLL020C
YKL129C	YLL019W-A
YKL120W	YLR002C
YKL112W	YLR023C
YKL110C	YLR056W
YKL103C	YLR154W-A
YKL101W	YLR154W-E
YKL099C	YLR154W-F
YKL088W	YLR154C-G
YKL074C	YLR156W
YKL063C	YLR157C-C

YKL035W	YLR161W
YKL029C	YLR174W
YKL028W	YLR185W
YKL004W	YLR222C
YKR010C	YLR222C-A
YKR027W	YLR249W
YKR029C	YLR257W
YKR039W	YLR285W
YKR051W	YLR298C
YKR064W	YLR299C-A
YKR066C	YLR300W
YKR067W	YLR302C
YKR098C	YLR308W
YLL056C	YLR330W
YLL041C	YLR333C
YLL032C	YLR338W
YLL021W	YLR339C
YLR003C	YLR341W
YLR017W	YLR354C
YLR034C	YLR376C
YLR044C	YLR399W-A
YLR066W	YLR406C
YLR083C	YLR428C
YLR102C	YLR437C
YLR135W	YLR447C
YLR139C	YLR462W
YLR150W	YLR464W
YLR214W	YLR466W
YLR223C	YLR466C-A
YLR224W	YLR467C-A
YLR225C	YML133W-A
YLR231C	YML129C
YLR244C	YML126C
YLR246W	YML116W
YLR265C	YML074C
YLR278C	YML073C
YLR283W	YML054C
YLR287C	YML054C-A
YLR297W	YML034C-A
YLR305C	YML027W
YLR320W	YML026C
YLR344W	YML025C
YLR372W	YML024W
YLR378C	YML009C

YLR389C	YML008C
YLR390W-A	YMR013C
YLR399C	snR75
YLR400W	snR74
YLR413W	snR73
YLR422W	YMR013W-A
YML121W	YMR015C
YML118W	YMR017W
YML110C	YMR030W-A
YML109W	YMR033W
YML103C	YMR050C
YML101C-A	YMR051C
YML059C	YMR052W
YML058W	YMR055C
YML043C	YMR056C
YML042W	YMR087W
YML038C	YMR102C
YML029W	YMR119W-A
YML010W	YMR134W
YML006C	YMR142C
YMR001C	YMR143W
YMR002W	YMR153C-A
YMR041C	YMR156C
YMR108W	YMR158W
YMR112C	YMR178W
YMR121C	YMR194C-B
YMR122W-A	YMR195W
YMR132C	YMR199W
YMR200W	YMR207C
YMR205C	YMR208W
YMR206W	YMR232W
YMR238W	YMR242W-A
YMR290C	YMR256C
YMR291W	YMR315W-A
YMR296C	YMR316C-B
YMR297W	YMR318C
YMR305C	YMR320W
YMR306W	YNL339W-B
YMR311C	YNL339W-A
YMR312W	YNL308C
YMR319C	YNL302C
YNL322C	YNL292W
YNL284C	YNL289W
YNL275W	YNL226W

YNL270C	YNL225C
YNL268W	YNL218W
YNL254C	YNL177C
YNL253W	YNL170W
YNL201C	YNL149C
YNL167C	YNL147W
YNL124W	YNL138W-A
YNL119W	YNL134C
YNL068C	YNL117W
YNL067W-B	YNL103W-A
YNL067W	YNL081C
YNL064C	YNL069C
YNL058C	YNL057W
YNL029C	YNL055C
YNL027W	YNL054W-B
YNR012W	YNL054W-A
YNR017W	YNL031C
YNR030W	YNL030W
YNR033W	YNL003C
YNR040W	YNR003W-A
YNR050C	YNR019W
YNR053C	YNR024W
YOL158C	YNR068C
YOL130W	YNR072W
YOL126C	YOL165C
YOL111C	YOL138C
YOL100W	YOL077W-A
YOL097C	YOL052C-A
YOL088C	YOL046C
YOL086C	YOL042W
YOL085W-A	YOL007C
YOL082W	YOR015W
YOL030W	YOR019W
YOL023W	YOR031W
YOL006C	YOR032C
YOL005C	YOR032W-A
YOL004W	YOR036W
YOR005C	YOR049C
YOR017W	YOR050C
YOR034C	YOR107W
snR62	YOR136W
YOR043W	YOR141C
YOR045W	YOR153W
YOR063W	YOR155C

YOR073W-A	YOR161W-B
YOR095C	YOR185C
YOR096W	YOR186W
YOR108W	YOR186C-A
YOR118W	YOR192C
YOR127W	YOR192C-C
YOR166C	YOR231W
snR36	YOR231C-A
YOR217W	YOR232W
YOR227W	snR17a
YOR233W	YOR273C
YOR254C	YOR298C-A
YOR287C	YOR299W
YOR316C-A	YOR312C
YOR318C	YOR314W
YOR319W	YOR315W
YOR329W-A	YOR364W
YOR336W	YOR366W
YOR354C	YOR369C
YOR355W	YOR371C
YOR358W	YOR388C
YOR362C	YPL283W-B
YOR370C	YPL283W-A
YOR373W	YPL274W
YOR387C	YPL257W-A
YOR390W	YPL257W-B
YOR394C-A	YPL224C
YPL283C	YPL172C
YPL279C	YPL144W
YPL277C	snR17b
YPL268W	YPL135C-A
YPL263C	YPL111W
YPL262W	YPL041C
YPL259C	YPL033C
YPL238C	YPL028W
YPL235W	YPL026C
YPL226W	YPL019C
YPL222C-A	YPR005C
YPL221W	YPR032W
YPL217C	YPR035W
YPL211W	YPR071W
YPL206C	YPR074W-A
YPL179W	YPR151C
YPL168W	YPR160C-A

YPL148C	YPR177C
YPL099C	YPR184W
YPL097W	YPR195C
YPL089C	
YPL088W	
YPL085W	
YPL083C	
YPL077C	
YPL072W	
YPL068C	
YPL066W	
YPL043W	
YPL037C	
YPL036W	
YPL024W	
YPR016C	
YPR018W	
YPR026W	
YPR029C	
YPR030W	
YPR050C	
YPR063C	
YPR099C	
YPR137W	
YPR143W	
YPR149W	
YPR167C	
YPR169W	
YPR196W	
YPR202W	

Table S2 ATP-dependent chromatin remodelers that correspond to the ordered columns in Figure 11B

ATP-dependent chromatin remodeler	reference	Pubmed ID
swi1_minimal_medium_experiment	Sudarsanam00	10725359
snf2_minimal_medium_experiment	Sudarsanam00	10725359
swi1_rich_medium_experiment	Sudarsanam00	10725359
snf2_rich_medium_experiment	Sudarsanam00	10725359
swr1	Meneghini03	12628191
swr1_del	Mizuguch04	14645854
ino80_del	Mizuguch04	14645854
ino80	Attikum04	15607975
arp8	Attikum04	15607975

ino80 + 0.1% MMS vs. ino80	Attikum04	15607975
arp8 + 0.1% MMS vs. arp8	Attikum04	15607975
SWC6 (VPS71)	Krogan04	15353583
SWR1	Krogan04	15353583
SWC2 (VPS72)	Krogan04	15353583
isw2	Fazzio01	11533234
isw2 c.i.	Fazzio01	11533234
isw2 rpd3	Fazzio01	11533234
isw2 c.i. rpd3 c.i.	Fazzio01	11533234
isw2 sin3	Fazzio01	11533234
isw2 ume6	Fazzio01	11533234
isw2 rpd3 vs. rpd3	Fazzio01	11533234
isw2 sin3 vs. sin3	Fazzio01	11533234
rsc3-2	Angus-Hill01	11336698
rsc30 null	Angus-Hill01	11336698
IES6	Hughes00	10929718
IOC4	Hughes00	10929718
ISW1	Hughes00	10929718
ISW1_ISW2	Hughes00	10929718
ISW2	Hughes00	10929718
ARP4	Mnaimneh04	15242642
RSC8	Mnaimneh04	15242642
MOT1-14	Dasgupta02	11880621
MOT1-42	Dasgupta02	11880621
